# The Psychological Burden of Vitiligo: Investigating the Depressive Symptoms in Patients with Vitiligo: A Case–Control Study

**DOI:** 10.3390/medicina61091589

**Published:** 2025-09-03

**Authors:** Amr Molla, Muayad Albadrani

**Affiliations:** 1 Department of Medicine, College of Medicine, Taibah University, Madinah 42353, Saudi Arabia; 2 Department of Family and Community Medicine and Medical Education, College of Medicine, Taibah University, Madinah 42353, Saudi Arabia; 3 Health and Life Research Center, Taibah University, Madinah 42353, Saudi Arabia

**Keywords:** depressive symptoms, PHQ-9, psychological impact, Saudi Arabia, VASI, vitiligo

## Abstract

*Background and Objectives*: Vitiligo is a chronic autoimmune skin disorder characterized by the loss of pigmentation, resulting in depigmented patches due to the destruction of melanocytes. This condition can lead to considerable psychological distress, and research indicates a possible connection with major depressive disorder (MDD). Nonetheless, the depth and nature of this association, particularly within the Saudi population, have not been thoroughly investigated. This case–control study seeks to explore the relationship between vitiligo and depressive symptomatology, evaluate the severity of depressive symptoms in vitiligo patients compared to control subjects, and examine the association between the clinical severity of vitiligo (assessed using the Vitiligo Area Scoring Index, VASI) and depressive symptoms (measured using the PHQ-9, a validated screening instrument based on DSM criteria). *Materials and Methods*: A total of 700 participants were included, comprising 340 individuals with vitiligo and 360 controls with other skin conditions. Participants completed a web-based questionnaire that collected sociodemographic data and included the PHQ-9 assessment. The severity of vitiligo was determined using the VASI. Statistical analysis involved using the computer program IBM Statistical Program for Social Sciences (SPSS) (version 26.0). *Results*: The average PHQ-9 score was significantly higher for patients with vitiligo (8.28 ± 7.36) compared to controls (6.30 ± 4.70, *p* = 0.028). While the overall rates of mild to severe depression were comparable (41.5% in vitiligo patients versus 40.3% in controls, *p* = 0.748), vitiligo patients exhibited higher occurrences of moderately severe depression (11.8%) and severe depression (10.9%) compared to controls (5.8% and 0.8%, respectively; *p* < 0.001). A weak, yet significant, positive correlation was found between VASI and PHQ-9 scores (ρ = 0.184, *p* < 0.001). The vulgaris and segmental types of vitiligo exhibited the highest median depression scores (PHQ-9: 11 and 9, respectively; *p* < 0.001). Logistic regression analysis indicated that those with genital vitiligo had greater odds of experiencing depression (OR = 12.10, *p* = 0.039), while those with universalis vitiligo faced even higher odds (OR ≈ 26,837.84, *p* = 0.001). Interestingly, higher VASI scores were linked to lower odds of depression (OR = 0.927, *p* = 0.029). Additionally, the risk of depression significantly increased with higher income levels and among individuals aged 50 years and older. *Conclusions*: Although the overall prevalence of depression was not significantly different between vitiligo patients and controls, the degree of depressive symptoms was notably more severe in those with vitiligo. Specific clinical subtypes, particularly genital and universalis vitiligo, were found to be more closely associated with an increased risk of depression. These results highlight the importance of regular mental health screenings and customized psychosocial support in dermatological care, especially for high-risk groups.

## 1. Introduction

Vitiligo is a chronic, immune-mediated, dermatological disorder marked by selective destruction of melanocytes, leading to sharply demarcated depigmented macules and patches across the skin [1]. As the skin serves not only a protective function but also an essential role in social and personal identity [2], visible skin conditions, such as vitiligo, often carry substantial psychological burdens. The aesthetic implications of the disease can significantly impact patients’ emotional well-being, frequently contributing to the onset or exacerbation of psychiatric conditions, particularly major depressive disorder (MDD) [1]. Furthermore, autoimmune comorbidities, like thyroid disorders, are common in vitiligo patients and may further compound psychological stress [1,2].

Vitiligo is increasingly recognized as a multifactorial disease involving genetic, immunological, and environmental determinants. In Saudi Arabia, where consanguineous marriages are culturally prevalent, there is a growing body of evidence indicating a strong genetic contribution to vitiligo susceptibility. Case–control and cross-sectional studies have shown significantly higher rates of parental consanguinity and familial aggregation among vitiligo patients compared to controls, with first-cousin marriages, in particular, conferring a markedly increased risk for both childhood- and adult-onset vitiligo [3,4]. These findings underscore the complex heritable component of vitiligo in consanguineous populations, reinforcing the need for targeted genetic counseling and population-specific disease prevention and management strategies.

MDD is characterized by persistent feelings of sadness, diminished interest or pleasure, and disturbances in sleep, appetite, and cognition, which can severely impair daily functioning [5]. In the context of vitiligo, MDD may arise not only from psychosocial distress but also from shared biological pathways involving immune dysregulation and systemic inflammation. Research suggests that both conditions may involve similar leukocyte signatures and inflammatory mediators, indicating a potential brain–skin inflammatory axis [6]. This psychosomatic overlap suggests a bidirectional relationship, where each condition can exacerbate the other, especially in individuals facing societal stigma and internalized distress.

Vitiligo has a profound psychosocial impact, often leading to emotional distress, social withdrawal, and reduced quality of life. Studies have shown that up to 80% of vitiligo patients experience depressive symptoms, with significantly higher rates of stress and anxiety compared to healthy controls [7]. The visibility of lesions, particularly on exposed or intimate areas, can intensify feelings of embarrassment, stigma, and low self-esteem [8,9]. Moreover, a moderate positive correlation has been observed between depression severity and quality of life impairment, suggesting that psychological distress in vitiligo is not merely reactive but may be deeply intertwined with disease perception and social functioning [7,8,9,10].

Empirical evidence consistently highlights the significant psychological burden associated with vitiligo. Studies by Nasser et al. (2021), Singla et al. (2023), and Kussainova et al. (2020) consistently show high prevalence rates of psychological distress among vitiligo patients, with reported depression rates ranging from 36% to 80%, anxiety from 35.8% to 78%, and stress from 32% to 76% [7,11,12]. Vernwal (2017) further demonstrated that psychiatric morbidity is significantly higher in individuals with vitiligo compared to healthy controls (62% vs. 25%) [13]. Several demographic and clinical factors have been identified as contributing to this increased burden, including female gender and extensive body surface area involvement [12,13]. Lesions on exposed areas are associated with higher psychiatric morbidity [13]. A recent cross-sectional study in Saudi Arabia further supports this association, reporting that over half of vitiligo patients met criteria for major depressive disorder, with specific vitiligo types and socioeconomic factors significantly influencing depression severity [14]. The psychological impact of vitiligo is comparable to other severe skin disorders, emphasizing the need for holistic care and collaboration with mental health professionals in managing vitiligo patients [11,12].

This research aims to evaluate the relationship between vitiligo and the presence and severity of depressive symptoms within a population in Saudi Arabia. Specifically, it investigates whether individuals with vitiligo are more vulnerable to experiencing depressive symptoms compared to those without the condition. Additionally, the study examines the relationship between the extent of vitiligo, as measured by the Vitiligo Area Scoring Index (VASI), which quantifies the severity of vitiligo in clinical research [15]. The VASI estimates the affected skin percentage by multiplying the area involved by the degree of depigmentation, along with the severity of depression, as measured using the Patient Health Questionnaire-9 (PHQ-9), a validated screening tool based on DSM criteria. While the study does not aim to determine causality or differentiation between biological and psychosocial mechanisms, its well-controlled design enables a more robust assessment of the association between vitiligo and depressive symptoms.

## 2. Materials and Methods

### 2.1. Study Design and Sample

This case–control study involved 700 participants, with 340 individuals diagnosed with vitiligo as the case group and a control group of 360 individuals without a vitiligo diagnosis. These participants were chosen using a simple random sampling technique from hospitals in various regions of Saudi Arabia. The case group patients were drawn from the databases of participating hospitals, covering a one-year period from January 2024 to December 2024.

#### 2.1.1. Inclusion Criteria

Individuals diagnosed with vitiligo, as measured by the VASI, are included in the study. Participants must have been aged 18 to 85 and residing in Saudi Arabia, and they should have been capable of giving informed consent and completing assessments. The VASI evaluates the extent and severity of vitiligo by assessing the affected body surface area and skin depigmentation. It divides the body into segments, scoring depigmentation from 0% (no depigmentation) to 100% (total depigmentation), providing a comprehensive assessment of the disease’s severity.

#### 2.1.2. Exclusion Criteria

Individuals with pre-existing psychiatric disorders such as bipolar disorder and schizophrenia, as well as those with other forms of skin depigmentation like albinism and chemical leukoderma, were excluded to minimize confounding effects and ensure that depressive symptoms assessed during the study were temporally associated with vitiligo or other dermatological conditions, rather than stemming from prior psychiatric morbidity.

Participants with cognitive impairments that could hinder their understanding of the questionnaire or informed consent process were also excluded, along with pregnant or lactating women, due to potential hormonal effects on skin conditions and emotional well-being. The control group consisted of non-vitiligo patients with various dermatological infections, including tinea, molluscum contagiosum, warts, and herpes, sourced from the same healthcare facilities as the case group for comparative analysis.

### 2.2. Data Collection

The majority of data was collected through an online questionnaire distributed via social media platforms, ensuring broad reach and accessibility in both the vitiligo and control groups. The other data was collected through in-clinic interviews and phone calls, focusing on sociodemographic details such as gender, age, marital status, nationality, occupation, and monthly income. The second section utilized the PHQ-9 score to assess the severity of depressive symptoms, serving as a clinician-administered tool that screens for depression based on the *Diagnostic and Statistical Manual of Mental Disorders, Fourth Edition* (DSM-IV) [16,17]. The PHQ-9 was selected due to its brevity, ease of administration, and strong psychometric validity across diverse populations. Unlike clinician-administered scales, such as the MADRS or HDRS, the PHQ-9 is a self-administered screening tool that facilitates efficient data collection in outpatient and community settings. The study’s independent variable was the type of vitiligo, classified into non-segmental forms (generalized, acrofacial, and universalis) and segmental vitiligo, along with unclassified cases. The dependent variable, representing the severity of MDD, was operationalized through PHQ-9 scores, with identified categories ranging as follows: 0 = none-minimal, 1 = mild, 2 = moderate, 3 = moderately severe, and 4 = severe depression. The binary depression variable was divided into two categories, indicating whether the patient has depression (mild to severe) or does not have depression (none–minimal). Covariates included gender, age, nationality, marital status, employment, and income levels. Income levels were categorized into four groups, ranging from less than USD 810 per month for the low-income group to over USD 5265 per month for the high-income group, with the moderately high-income group (ranging from USD 2430 to USD 5265 per month) used as reference in the regression analysis. Marital status was classified as single, married, or divorced, with married participants considered the reference group. Job status was divided into the following three categories: employed, unemployed, or student.

### 2.3. Data Analysis

The data were entered into an Excel sheet for review and then transferred to SPSS (Statistical Package for the Social Sciences) version 26 for analysis. Descriptive analysis was performed to describe categorical data using numbers and percentages. Numerical variables were described using the mean and standard deviation if they were normally distributed and the median and interquartile range (IQR) if not. Fisher’s Exact test was utilized to evaluate the relationship between categorical variables. The Mann–Whitney test was used for binary depression, and the Kruskal–Wallis test was used for depression severity. Logistic regression analysis was performed to calculate the adjusted odds ratios (aORs). The final regression model was adjusted for age, gender, nationality, parental consanguinity, and family history of vitiligo. A *p*-value of less than 0.05 was considered to be statistically significant.

### 2.4. Ethical Considerations

The study received official approval from the Institutional Review Board (IRB) of Taibah, with reference number TU-039-22, on 5 June 2023. Informed consent was acquired from participants before they filled out the questionnaire. Additionally, all participants were informed that the data gathered from this questionnaire would be used solely for research purposes and would be maintained as confidential records.

## 3. Results

**Figure 1:**
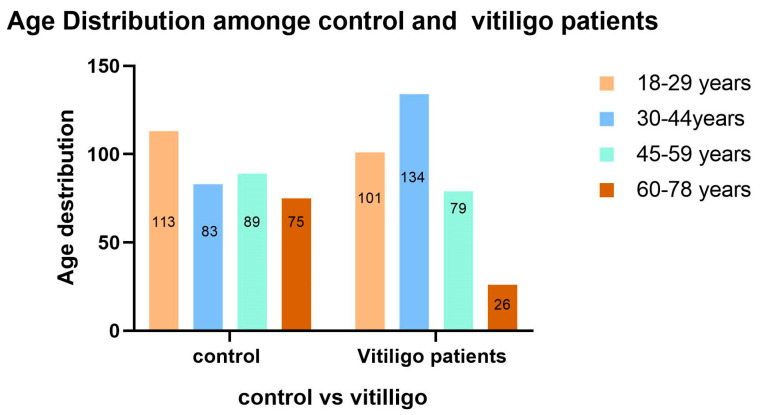
Age distribution among case and control groups (*n* = 700).

**Figure 2:**
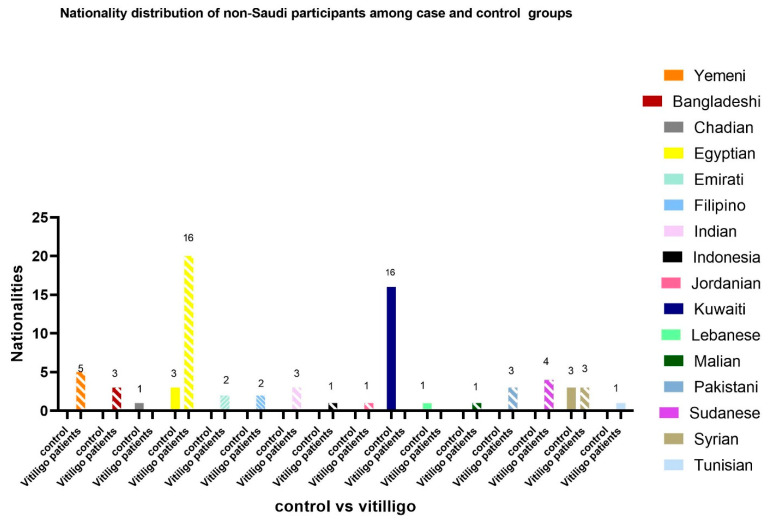
Nationality distribution of non-Saudi participants among case and control groups (*n* = 75).

Out of 700 participants, 49.3% (*n* = 345) were female and 50.7% (*n* = 355) were male, with no significant gender difference between vitiligo cases and controls (*p* = 0.080). The mean age of vitiligo patients was 38.18 years (SD ± 13.63), which was significantly lower than the control group’s mean age of 41.65 ± 16.13 years (*p* = 0.009) (Figure 1). Most participants were Saudi nationals (*n* = 626; 89.4%), with a higher proportion of non-Saudis found among vitiligo cases (14.7%) compared to controls (6.7%) (*p* = 0.001). Non-Saudi individuals were more prevalent among the vitiligo group (50/340; 14.7%), with Egyptians being the most represented, compared to controls (24/360; 6.7%), with Kuwaitis being the most prevalent (Figure 2).

Regarding marital status, 50.9% (*n* = 356) were married, 38.7% (*n* = 271) were single, 8.6% (*n* = 60) were divorced, and 1.9% (*n* = 13) were widowed. Singles were more common among vitiligo patients (45%) than controls (32.8%), while marriage was more common in controls (58.3%) (*p* < 0.001). Employment was reported in 41.4% (*n* = 290), unemployment in 44.6% (*n* = 312), and student status in 14.0% (*n* = 98), with significantly higher unemployment in vitiligo patients (53.8%) compared to controls (35.8%) (*p* < 0.001). The majority (*n* = 348; 49.7%) had a low monthly income below $810 USD, which was more prevalent among vitiligo patients (74.4%) than controls (26.4%) (*p* < 0.001). All details are shown in Table 1.

Table 2 shows the depression findings, with a mean PHQ-9 score that was higher in vitiligo patients at 8.28 (SD ± 7.36) compared to 6.30 (SD ± 4.70) in controls (*p* = 0.028). Regarding depression severity, 41.2% (*n* = 140) of vitiligo patients had none-to-minimal depression, compared to 40.3% (*n* = 145) of controls. Mild depression was more common in controls (35.3%; *n* = 127) than in cases (18.2%; *n* = 62). Moderate depression was nearly equal between cases (17.9%; *n* = 61) and controls (17.8%; *n* = 64). However, moderately severe depression (11.8%; *n* = 40) and severe depression (10.9%; *n* = 37) were more prevalent among vitiligo patients compared to controls (5.8%, *n* = 21 and 0.8%, *n* = 3, respectively), with a statistically significant difference in severity distribution (*p* < 0.001).

Table 3 shows the type and severity among vitiligo patients and their relation with the PHQ-9 score. The most common type was acrofacial (48.5%; *n* = 165), followed by vulgaris (21.5%; *n* = 73), focal (19.1%; *n* = 65), genital (4.7%; *n* = 16), universalis (3.2%; *n* = 11), and segmental (2.9%; *n* = 10). The VASI score, measuring vitiligo extent, had a median (IQR) of 5 out of 100 (9), with scores ranging from 1 to 92.

A weak, positive, significant correlation was found between VASI and PHQ-9 scores (ρ = 0.184, *p* < 0.001), highlighting that greater vitiligo severity is associated with higher depression levels. Additionally, a statistically significant difference was observed between depression scores across vitiligo types, where the highest median (IQR) PHQ-9 score was in vulgaris patients at 11 (15), followed by segmental at 9 (17) (*p* < 0.001).

Table 4 demonstrates the impact of depression severity on VASI scores among vitiligo patients. A statistically significant difference in VASI scores across different depression severity groups (*p* = 0.033) was found. The median (IQR) VASI scores are likely to increase as depression severity worsens, ranging from 5 (9) in the none-to-minimal depression category to 9.5 (13) in the moderately severe category. Individuals experiencing severe depression had a median (IQR) VASI score of 8 (12). Additionally, the binary depression category showed that vitiligo patients with mild-to-severe depression had significantly higher VASI scores compared to those without or having minimal depression (median: 7 vs. 5; *p* = 0.015).

Table 5 investigates the relationship between variables and their impact on depression among vitiligo patients. Statistical significance was found in factors including age, income, VASI score, and vitiligo type. Patients aged 18–29 years (OR = 0.132, 95% CI: 0.044–0.398, *p* < 0.001) and 30–49 years (OR = 0.146, 95% CI: 0.064–0.333, *p* < 0.001) had significantly lower odds of depression compared to those aged 50 years or more. Higher income was associated with increased odds of depression, with those earning USD 2430–5264 (OR = 9.504, 95% CI: 2.693–33.543, *p* < 0.001) and USD 810–2429 (OR = 4.510, 95% CI: 1.410–14.431, *p* = 0.011) compared to the lowest income group. Additionally, a higher VASI score was significantly associated with decreasing odds of depression (OR = 0.927, 95% CI: 0.867–0.992, *p* = 0.029). Regarding vitiligo types, genital vitiligo (OR = 12.097, 95% CI: 1.133–129.224, *p* = 0.039) and universalis vitiligo (OR = 26,837.836, 95% CI: 82.925–8,685,810.081, *p* = 0.001) were associated with markedly higher odds of depression compared to segmental vitiligo.

Additional sociodemographic predictors of depression across the full sample are provided in Supplementary Table S1. Several factors were significantly associated with depression. Female participants had lower odds of depression compared to males (OR = 0.661, 95% CI: 0.460–0.949, *p* = 0.025). Younger age groups showed significantly reduced odds of depression compared to those aged 50 years and older, with odds ratios of 0.237 (95% CI: 0.124–0.453, *p* < 0.001) for the 18–29 age group and 0.326 (95% CI: 0.211–0.502, *p* < 0.001) for the 30–49 group. Moreover, unemployed individuals had lower odds of depression compared to students (OR = 0.476, 95% CI: 0.248–0.914, *p* = 0.026). Regarding income, participants earning USD 2430–5264 (OR = 2.512, 95% CI: 1.408–4.482, *p* = 0.002) and USD 810–2429 (OR = 1.998, 95% CI: 1.151–1.998, *p* = 0.014) had higher odds of depression than those earning less than USD 810. Lastly, participants who experienced vitiligo had significantly lower odds of depression compared to those without vitiligo (OR = 0.500, 95% CI: 0.328–0.762, *p* = 0.001).

Further analysis of demographic associations with VASI scores is detailed in Supplementary Table S2, demonstrating 34% of the variance in VASI scores among vitiligo patients could be explained by gender, nationality, age group, marital status, job status, income level, and binary depression status using linear regression analysis (R^2^ = 0.34). None of the factors showed a statistically significant association with VASI scores.

Variance Inflation Factor (VIF) analysis indicates no evidence of problematic multicollinearity among predictors (all VIFs < 2.0; tolerances > 0.50). Condition indices were <30, with no clustering of high variance proportions, suggesting a stable coefficient estimate.

## 4. Discussion

This case–control study, which included 700 participants, provides valuable insights into the connection between vitiligo and depression, specifically concerning the severity of depressive symptoms. Although the overall rates of depression (ranging from mild to severe) were comparable between vitiligo patients (41.5%) and the control group (40.3%, *p* = 0.748), the distribution of severity levels showed significant differences. A notably larger percentage of vitiligo patients experienced moderately severe (11.8%) and severe depression (10.9%) compared to controls (5.8% and 0.8%, respectively), with a statistically significant difference in severity distribution (*p* < 0.001). This finding aligns with the results from Molla et al. (2024), who reported that 10.9% of vitiligo patients had severe depression, suggesting that vitiligo is associated with more intense depressive symptoms rather than a higher overall prevalence [14].

These results are supported by previous studies, including the work of Karia et al. (2015) [18], who reported that 30% of vitiligo patients suffered from psychiatric disorders, particularly depression (20%) and anxiety (8%). Their study also demonstrated that vitiligo patients have significantly poorer quality of life scores, correlating with both psychological distress and body surface area involvement. Research indicates that 34% of individuals with vitiligo experience psychiatric conditions, with 12% suffering from anxiety and 22% from depression [19]. A different assessment revealed a psychiatric morbidity prevalence of 16.2% among vitiligo patients, with depression occurring in 10% and anxiety in 3.3% [20]. In a study conducted in India, approximately 34% of vitiligo patients exhibited psychiatric issues, including adjustment disorder (56%), depressive episodes (22%), and dysthymia (9%) [21]. Another investigation involving 180 vitiligo patients reported a clinical prevalence of psychiatric morbidity at 48% [22]. Additionally, research by Ahmed et al. [23] identified psychiatric comorbidity in 42% of patients, with major depression and anxiety disorders being the predominant conditions. Furthermore, a study conducted by Rashid et al. [24] found a psychiatric comorbidity rate of 24%. These findings are comparable to the results of our own study. Similarly, a systematic review and meta-analysis by Lai et al. (2017) [25] confirmed the increased risk of depression in vitiligo patients, revealing a pooled prevalence of 25.3% and a fivefold increased likelihood of depression compared to controls. These findings suggest that, while the overall prevalence may vary depending on methodology, the psychological burden of vitiligo is both substantial and consistent across populations.

The demographic and socioeconomic characteristics of patients with vitiligo demonstrate a significant correlation with the risk of depression when compared to control groups. Notably, patients with vitiligo are, on average, younger, with a mean age of 38.18 years compared to 41.65 years for the controls (*p* = 0.009). This difference in age may suggest varying experiences and coping mechanisms between the two groups. Moreover, individuals with vitiligo face higher unemployment rates; 53.8% of them are unemployed, in contrast to only 35.8% of the controls (*p* < 0.001). This disparity in employment may contribute to financial stress, which is a known risk factor for depression. Additionally, a striking 74.4% of patients with vitiligo earn less than USD 810 per month, compared to just 26.4% of the control group (*p* < 0.001), further highlighting the economic challenges faced by these individuals. Regression analysis reveals that younger patients, specifically those aged 18–29 and 30–49, show significantly lower odds of experiencing depression than their counterparts over 50 years old, with odds ratios of 0.132 and 0.146, respectively (*p* < 0.001). This finding aligns with previous studies by Salman et al. and Molla et al. [9,14], suggesting that younger patients may possess better coping strategies or may be affected by shorter disease duration. Altogether, these comparisons underscore the complex interplay of age, employment status, and income level in relation to the mental health challenges faced by individuals with vitiligo.

The association between sociodemographic factors and depression was further echoed in the findings by Alharbi (2020) [26], who noted that moderate-to-severe depressive symptoms were more common among children, adolescents, single individuals, and those with lower education or shorter disease duration. Additionally, Alharbi identified phototherapy as a factor linked to increased depressive symptoms, which may reflect patients’ perception of disease severity or treatment burden. Interestingly, gender and lesion distribution were not significant predictors of depression in that study, emphasizing the complex interplay of personal and clinical factors.

The subtype of vitiligo has a significant influence on psychological outcomes. The vulgaris and segmental types exhibited the highest PHQ-9 scores (median = 11 and 9), whereas universalis had the lowest score (median = 1), despite its clinical severity. Logistic regression analysis indicated that genital (OR = 12.1, *p* = 0.039) and universalis vitiligo (OR = 26,837.8, *p* = 0.001) were linked to substantially increased odds of depression compared to the segmental variant. These findings highlight that the location and pattern of skin involvement, particularly in socially or sexually sensitive areas, might have a more significant effect on mental health than the overall extent of the disease. This trend is consistent with findings by Nguyen et al. (2016), who noted a strong correlation between intimate and facial lesions and depression and anxiety due to increased stigma and concerns about body image [8].

Despite finding a weak, positive correlation between the severity of vitiligo, as measured by the VASI score, and the PHQ-9 score (ρ = 0.184, *p* < 0.001), regression analysis revealed an unexpected relationship. Higher VASI scores were associated with lower odds of depression (OR = 0.927, *p* = 0.029). This counterintuitive finding may suggest that individuals psychologically adapt to more extensive vitiligo or that statistical outliers, such as those with universal vitiligo, are influencing the results. Similarly, Molla et al. noted that extensive vitiligo might lead to lower psychological distress, possibly due to a reduced contrast between the affected and unaffected areas of skin [14]. In light of the absence of multicollinearity, the associations observed, particularly the unexpected inverse relationship between VASI and depressive symptoms, are unlikely to result from statistical distortion caused by overlapping predictor variables.

The strengths of this study include a large and appropriately matched sample, the use of validated assessment tools, and comprehensive multivariate analysis to control for confounding variables. Additionally, it provides culturally relevant insights from a Middle Eastern perspective, offering valuable information to a field that is often underexplored.

The case–control design of the study limits causal inferences between vitiligo and depression. Reliance on self-reported measures like PHQ-9 and VASI may introduce bias, especially in stigmatized environments. Recruitment from clinical settings may affect generalizability, and small sample sizes for less common subtypes like genital and universalis vitiligo reduce statistical power. The exclusion of participants with prior psychiatric diagnoses and the absence of onset data limit the ability to explore temporal and bidirectional relationships between vitiligo and depression. Future research should consider longitudinal designs and broader inclusion criteria to better understand the complex interplay between dermatological and psychiatric conditions. Future research should adopt longitudinal approaches, include larger cohorts of these subtypes, and investigate biological markers, psychosocial interventions, and qualitative aspects of stigma and coping to develop targeted care strategies.

## 5. Conclusions

This study revealed that the overall prevalence of depression in vitiligo patients is similar to that of the general population, but the severity of depressive symptoms is significantly higher. Factors like clinical subtype, lesion location, and socioeconomic status contribute to psychological distress more than the disease’s extent. These findings highlight the need for comprehensive care that includes mental health support, especially for those more susceptible to psychosocial stressors. Targeted mental health interventions are essential to address the psychological impacts of vitiligo. Moreover, multicollinearity diagnostics confirmed the stability of the regression model, reinforcing the validity of the observed associations between clinical factors and depressive symptoms.

## Figures and Tables

**Table 1:** Sociodemographic characteristics of case and control participants (*n* = 700).

Factor	Category	Vitiligo Patients (*n* = 340) *n* (%)	Controls (*n* = 360) *n* (%)	Total (*n* = 700) *n* (%)	*p*-Value
Age (years)	Mean ± SD	38.18 ± 13.630	41.65 ± 16.127	39.96 ± 15.056	0.009
	Median (IQR)	36 (22)	42 (28)	37 (60)	
	Min-Max	18–76	18–78	18–78	
Gender	Female	156 (45.9)	189 (52.5)	345 (49.3)	0.080
	Male	184 (54.1)	171 (47.5)	355 (50.7)	
Nationality	Saudi	290 (85.3)	336 (93.3)	626 (89.4)	0.001
	Non-Saudi	50 (14.7)	24 (6.7)	74 (10.6)	
Marital status	Single	153 (45)	118 (32.8)	271 (38.7)	<0.001
	Married	146 (42.9)	210 (58.3)	356 (50.9)	
	Divorced	41 (12.1)	19 (5.3)	60 (8.6)	
	Widowed	0 (0)	13 (3.6)	13 (1.9)	
Job status	Employed	128 (37.6)	162 (45)	290 (41.4)	<0.001
	Unemployed	183 (53.8)	129 (35.8)	312 (44.6)	
	Student	29 (8.5)	69 (19.2)	98 (14.0)	
Monthly Income	High	12 (3.5)	60 (16.7)	72 (10.3)	<0.001
	High-moderate	43 (12.6)	104 (28.9)	147 (21)	
	Low-moderate	32 (9.4)	101 (28.1)	133 (19)	
	Low	253 (74.4)	95 (26.4)	348 (49.7)	

**Table 2:** PHQ-9 depression scores and severity among participants (*n* = 700).

Factor	Category	Vitiligo Patients (*n* = 340)	Controls (*n* = 360)	Total (*n* = 700)	*p*-Value
PHQ-9 score	Mean ± SD	8.28 ± 7.361	6.30 ± 4.698	7.26 ± 6.211	0.028
	Median (IQR)	7 (13)	6 (6)	6 (9)	
	Min–Max	0–25	0–22	0–25	
Depression severity	None–minimal	140 (41.2)	145 (40.3)	285 (40.7)	<0.001
	Mild	62 (18.2)	127 (35.3)	189 (27.0)	
	Moderate	61 (17.9)	64 (17.8)	125 (17.9)	
	Moderately severe	40 (11.8)	21 (5.8)	61 (8.7)	
	Severe depression	37 (10.9)	3 (0.8)	40 (5.7)	
Binary Depression	None–minimal	140 (41.2)	145 (40.3)	285 (40.7)	0.809
	Yes (mild-to-severe)	200 (58.8)	215 (59.7)	415 (59.3)	

**Table 3:** Clinical Characteristics of Vitiligo Patients and their relation with PHQ-9 Score among the case group (*n* = 340).

Factor	Category	N (%)	PHQ-9 Median (IQR)	*p*-Value (Vitiligo Type)
Vitiligo Type	Acrofacial	165 (48.5)	7 (12)	<0.001
	Vulgaris	73 (21.5)	11 (15)	
	Focal	65 (19.1)	4 (9)	
	Genital	16 (4.7)	2.5 (8)	
	Universalis	11 (3.2)	1 (0)	
	Segmental	10 (2.9)	9 (17)	
VASI Score	Mean ± SD: 10.4 ± 14.672		Spearman’s ρ: 0.184	<0.001
	Median (IQR): 5 (9)			
	Min–Max: 1–92			

**Table 4:** Impact of depression severity on VASI Score among vitiligo patients (*n* = 340).

Factor	Category	VASI Score Median (IQR)	*p*-Value
Depression severity	None–minimal	5 (9)	0.033
	Mild	5 (8)	
	Moderate	8 (10)	
	Moderately severe	9.5 (13)	
	Severe depression	8 (12)	
Binary Depression	None–minimal	5 (9)	0.015
	Yes (Mild-to-severe)	7 (9)	

The Mann–Whitney test was used for binary depression, and the Kruskal–Wallis test was used for depression severity.

**Table 5:** Logistic regression analysis of depression with demographic and clinical predictors among vitiligo patients (*n* = 340).

Variable	Category	Odds Ratio	95% Confidence Interval		*p*-Value
			Lower	Upper	
Gender (Ref = Male)	Female	0.716	0.377	1.360	0.308
Nationality (Ref = Non-Saudi)	Saudi	0.714	0.270	1.884	0.496
Age (Ref ≥ 50)	18–29	0.132	0.044	0.398	<0.001
	30–49	0.146	0.064	0.333	<0.001
Marital status (Ref = Divorced/Widowed)	Single	2.057	0.628	6.738	0.233
	Married	2.650	0.835	8.412	0.098
Job (Ref = Student)	Employed	1.072	0.275	4.173	0.920
	Unemployed	0.608	0.199	1.857	0.382
Monthly Income (Ref = Low)	High	4.946	0.924	26.487	0.062
	High–moderate	9.504	2.693	33.543	<0.001
	Low–moderate	4.510	1.410	14.431	0.011
VASI score		0.927	0.867	0.992	0.029
Vitiligo type (Ref = Segmental)	Acrofacial	2.312	0.338	15.834	0.393
	Vulgaris	3.620	0.459	28.515	0.222
	Focal	6.160	0.829	45.754	0.076
	Genital	12.097	1.133	129.224	0.039
	Universalis	26,837.836	82.925	8,685,810.081181	0.001

## Data Availability

Data are available from the corresponding author upon reasonable request.

## Supplementary Materials

The following supporting information can be downloaded at: https://www.mdpi.com/article/10.3390/medicina61091589/s1, Table S1: Logistic Regression Analysis of depression with demographic factors and vitiligo occurrence (*n* = 700); Table S2: Linear regression analysis of VASI scores among Vitiligo Patients (*n* = 340).

## References

[1] Wolff K., Goldsmith L.A., Katz S.I., Gilchrest B.A., Paller A.S., Leffell D.J. (2008). Fitzpatrick’s dermatology in general medicine, 2 volumes. Transplantation.

[2] Simons R.E., Zevy D.L., Jafferany M. (2020). Psychodermatology of vitiligo: Psychological impact and consequences. Dermatol. Ther..

[3] Alharbi Y., Alrehaili Y., Alayoubi A.M. (2024). Impact of consanguinity and familial aggregation on vitiligo epidemiology in Saudi Arabia: A Case-control study. Cureus.

[4] Molla A., Alayoubi A.M., Jannadi R. (2024). First cousin marriages and the risk of childhood-onset vitiligo: Exploring the genetic background: A cross-sectional study. Clin. Cosmet. Investig. Dermatol..

[5] Vallerand I.A., Lewinson R.T., Parsons L.M., Hardin J., Haber R.M., Lowerison M.W., Barnabe C., Patten S.B. (2019). Vitiligo and major depressive disorder: A bidirectional population-based cohort study. J. Am. Acad. Dermatol..

[6] Otte C., Gold S.M., Penninx B.W., Pariante C.M., Etkin A., Fava M., Mohr D.C., Schatzberg A.F. (2016). Major depressive disorder. Nat. Rev. Dis. Primers.

[7] Nasser M.A., Raggi El Tahlawi S.M., Abdelfatah Z.A., Soltan M.R. (2021). Stress, anxiety, and depression in patients with vitiligo. Middle East Curr. Psychiatry.

[8] Nguyen C.M., Beroukhim K., Danesh M.J., Babikian A., Koo J., Leon A. (2016). The psychosocial impact of acne, vitiligo, and psoriasis: A review. Clin. Cosmet. Investig. Dermatol..

[9] Salman A., Kurt E., Topcuoglu V., Demircay Z. (2016). Social anxiety and quality of life in vitiligo and acne patients with facial involvement: A cross-sectional controlled study. Am. J. Clin. Dermatol..

[10] Silpa-archa N., Pruksaeakanan C., Angkoolpakdeekul N., Chaiyabutr C., Kulthanan K., Ratta-apha W., Wongpraparut C. (2020). Relationship Between Depression and Quality of Life Among Vitiligo Patients: A Self-assessment Questionnaire-based Study. Clin. Cosmet. Investig. Dermatol..

[11] Singla S., Malik Y.K., Dayal S., Gupta R. (2025). Quality of life, depression, anxiety, stress symptoms, and its association with vitiligo extent and distribution: A cross-sectional study. J. Neurosci. Rural. Pract..

[12] Kussainova A., Kassym L., Akhmetova A., Glushkova N., Sabirov U., Adilgozhina S., Tuleutayeva R., Semenova Y. (2020). Vitiligo and anxiety: A systematic review and meta-analysis. PLoS ONE.

[13] Vernwal D. (2017). A study of anxiety and depression in Vitiligo patients: New challenges to treat. Eur. Psychiatry.

[14] Molla A., Jannadi R., Alayoubi H., Altouri H., Balkhair M., Hafez D. (2024). Assessing the relationship between vitiligo and major depressive disorder severity: Cross-sectional study. JMIR Dermatol..

[15] Komen L., Da Graça V., Wolkerstorfer A., De Rie M.A., Terwee C.B., Van Der Veen J.P. (2015). Vitiligo Area Scoring Index and Vitiligo European Task Force assessment: Reliable and responsive instruments to measure the degree of depigmentation in vitiligo. Br. J. Dermatol..

[16] Kroenke K., Spitzer R.L., Williams J.B. (2001). The PHQ-9: Validity of a brief depression severity measure. J. Gen. Intern. Med..

[17] AlHadi A.N., AlAteeq D.A., Al-Sharif E., Bawazeer H.M., Alanazi H., AlShomrani A.T., Shuqdar R.M., AlOwaybil R. (2017). An arabic translation, reliability, and validation of Patient Health Questionnaire in a Saudi sample. Ann. Gen. Psychiatry.

[18] Karia S., Sousa A.D., Shah N., Sonavane S., Bharati A. (2015). Psychological morbidity in vitiligo—A case control study. Pigment. Disord..

[19] Saleh H.M., Salem S.A., El-Sheshetawy R.S., El-Samei A.M. (2008). Comparative study of psychiatric morbidity and quality of life in psoriasis, vitiligo and alopecia areata. Egypt. Dermatol. Online J..

[20] Sharma N., Koranne R.V., Singh R.K. (2001). Psychiatric morbidity in psoriasis and vitiligo: A comparative study. J. Dermatol..

[21] Mattoo S.K., Handa S., Kaur I., Gupta N., Malhotra R. (2001). Psychiatric morbidity in vitiligo and psoriasis: A comparative study from India. J. Dermatol..

[22] Rodríguez-Martín M., Melián D.D., Rodríguez M.S., Cabrera A., Bustínduy M.G. (2012). Assessment of Psychiatric Disorders in Vitiligo. Considerations for Our Daily Practice. Depression.

[23] Ahmed I., Ahmed S., Nasreen S. (2007). Frequency and pattern of psychiatric disorders in patients with vitiligo. J. Ayub Med. Coll. Abbottabad.

[24] Rashid M.H., Mullick M.S., Jaigirdar M.Q., Ali R., Nirola D.K., Salam M.A., Ahsan M.S. (2011). Psychiatric morbidity in psoriasis and vitiligo in two tertiary hospitals in Bangladesh. Bangabandhu Sheikh Mujib Med. Univ. J..

[25] Lai Y.C., Yew Y.W., Kennedy C., Schwartz R.A. (2017). Vitiligo and depression: A systematic review and meta-analysis of observational studies. Br. J. Dermatol..

[26] Alharbi M.A. (2020). Identifying patients at higher risk of depression among patients with vitiligo at outpatient setting. Mater. Socio-Medica.

