# Supplementary material for: The Psychological Burden of Vitiligo: Investigating the Depressive Symptoms in Patients with Vitiligo: A Case–Control Study
Source: Medicina (Kaunas). 2025 Sep 3;61(9):1589. doi: 10.3390/medicina61091589 (PMC12471397; doi:10.3390/medicina61091589)
Supplement: Supplementary file 1 [file medicina-61-01589-s001.zip › medicina-3819052-supplementary.pdf]

Supplementary Material: The Psychological Burden of Vitiligo: Investigating the Relationship with Major Depressive Disorder: A Case-Control Study

Supplementary Table S1: **Logistic Regression Analysis of depression with demographic factors and vitiligo occurrence (n=700)**

**Variable**	**Category**	**Odds Ratio**	**95% Confidence Interval**		**P value**
			**Lower**	**Upper**	
**Gender** (Ref = Male)	Female	0.661	0.460	0.949	**0.025**
**Nationality** (Ref = Non-Saudi)	Saudi	0.887	0.492	1.598	0.689
**Age** (Ref = ≥50)	18-29	0.237	0.124	0.453	**<0.001**
	30-49	0.326	0.211	0.502	**<0.001**
**Marital status** (Ref = Divorced/Widowed)	Single	0.931	0.474	1.826	0.834
	Married	1.254	0.711	2.213	0.434
**Job** (Ref=Student)	Employed	0.720	0.353	1.467	0.365
	Unemployed	0.476	0.248	0.914	**0.026**
**Monthly Income** (Ref=<810)	≥5265	1.667	0.815	3.406	0.161
	2430-5264	2.512	1.408	4.482	**0.002**
	810-2429	1.998	1.151	3.467	**0.014**
**Having vitiligo** (Ref=Yes)	No	0.500	0.328	0.762	**0.001**

***Supplementary Table S2*: Linear regression analysis of VASI scores among Vitiligo Patients (n=340)**

**Factors**	**Unstandardized Coefficient B**	**95% Confidence Interval**	**p-value**
Gender	0.165	[-3.275, 3.605]	0.925
Nationality	-3.332	[-8.446, 1.783]	0.201
Age	0.901	[-1.783, 3.584]	0.510
Marital Status	-0.579	[-3.271, 2.112]	0.672
Job Status	3.027	[-0.543, 6.598]	0.096
Income Level	-0.112	[-2.576, 2.352]	0.929
Binary Depression	-3.418	[-7.109, 0.272]	0.069
This model explains 34% of the variance in VASI scores ($R^2 = 0.34$)			
